# Toxicological Profile of PM from Different Sources in the Bronchial Epithelial Cell Line BEAS-2B

**DOI:** 10.3390/toxics11050413

**Published:** 2023-04-26

**Authors:** Gloria Melzi, Emma Nozza, Maria Agostina Frezzini, Silvia Canepari, Roberta Vecchi, Llorenç Cremonesi, Marco Potenza, Marina Marinovich, Emanuela Corsini

**Affiliations:** 1 Department of Pharmacological and Biomolecular Sciences, Università degli Studi di Milano, Via Balzaretti 9, 20133 Milan, Italy; gloria.melzi@unimi.it (G.M.);; 2 PhD Program in Experimental Medicine, Università degli Studi di Milano, Via L. Vanvitelli 32, 20129 Milan, Italy; 3 Department of Clinical Sciences and Community Health, Università degli Studi di Milano, Via della Commenda 19, 20122 Milan, Italy; 4 Department of Environmental Biology, Sapienza University of Rome, Via C. De Lollis 21, 00185 Rome, Italy; 5 Department of Physics, Università degli Studi di Milano, Via Celoria 16, 20133 Milan, Italy

**Keywords:** diesel exhaust particle, coke dust, incinerator ash, pellet ash, brake dust, genotoxicity, oxidative stress, BEAS-2B, in vitro

## Abstract

The toxicity of particulate matter (PM) is strictly associated with its physical-chemical characteristics, such as size or chemical composition. While these properties depend on the origin of the particles, the study of the toxicological profile of PM from single sources has rarely been highlighted. Hence, the focus of this research was to investigate the biological effects of PM from five relevant sources of atmospheric PM: diesel exhaust particles, coke dust, pellet ashes, incinerator ashes, and brake dust. Cytotoxicity, genotoxicity, oxidative, and inflammatory response were assessed in a bronchial cell line (BEAS-2B). BEAS-2B cells were exposed to different concentrations (25, 50, 100, and 150 μg/mL medium) of particles suspended in water. The exposure lasted 24 h for all the assays performed, except for reactive oxygen species, which were evaluated after 30 min, 1 h, and 4 h of treatment. The results showed a different action of the five types of PM. All the tested samples showed a genotoxic action on BEAS-2B, even in the absence of oxidative stress induction. Pellet ashes seemed to be the only ones able to induce oxidative stress by boosting the formation of reactive oxygen species, while brake dust resulted in the most cytotoxic. In conclusion, the study elucidated the differential response of bronchial cells to PM samples generated by different sources. The comparison could be a starting point for a regulatory intervention since it highlighted the toxic potential of each type of PM tested.

## 1. Introduction

Air pollution represents a recognized threat to human health and, among its components, particulate matter (PM) is considered one of the most important pollutants [1]. PM is constituted by a mixture of solid and liquid particles suspended in the atmosphere. PM can originate from different sources, and consequently can vary in composition, shape, and size distribution [2]. Typical PM components are inorganic ions (e.g., ammonium, sulfate, and nitrate), elemental and organic carbon (EC and OC, respectively), mineral dust and sea salt particles, heavy metals, and polycyclic aromatic hydrocarbons (PAHs) [3,4]; their relevance in PM samples depends on the emission sources and varies with particle size distribution [5].

PM sources can be divided into natural and anthropogenic, with the former accounting for about one order of magnitude more than the other on the global scale [6] but the latter generating the majority of smaller particles (e.g., with an aerodynamic diameter less than 2.5 µm) thus promoting a stronger impact on the human body. Some studies have been published focusing on the impact of different PM sources on human health [7,8], as well as on the environment [9,10]. In urban areas, PM from vehicular traffic comprising both exhaust and non-exhaust emissions [11], results highly hazardous [12]; during wintertime, PM emitted by residential heating using wood and pellet burning is increasing worldwide and its biological effects have been also assessed [9,13,14,15]. PM emissions from refinery and incinerator plants must be also taken into account for their potential effects on human health [16,17]. Indeed, all these sources release into the environment high quantities of pollutants comprising PAHs, heavy metals, and other toxic elements.

The respiratory tract is the first apparatus facing PM adverse effects. Here, larger particles (>2.5 µm) affect the upper respiratory tract, including the oral cavity, trachea, and bronchi; while smaller particles can diffuse to bronchioles and alveoli causing multiple adverse reactions [18]. PM inhalation can induce remodeling and loss of defensive barriers, e.g., cilia and mucociliary epithelium, or cellular damages [12]. Initially, PM deposition induces oxidative stress and the production of pro-inflammatory mediators [13,14,19,20,21]. Later, the onset of DNA damage and their possible misrepair can lead to mutations [22]. Exposure and inhalation of high levels of PM are associated with an increase in morbidity and mortality, increase in emergency room visits, hospital admission, and to the development of several diseases, such as cardiovascular diseases, respiratory and pulmonary diseases (such as chronic obstructive pulmonary diseases), non-communicable diseases, and premature death [14,23,24]. For these reasons, the International Agency for Research on Cancer (IARC) has classified outdoor PM_2.5_ as a carcinogen to humans [25].

The purpose of this study was to compare the effects of PM obtained from different relevant sources investigating cytotoxicity, oxidative stress, inflammation, and genotoxicity. As an experimental model, BEAS-2B cells were used. Cells were exposed to five types of PM characterized by very different chemical composition: diesel exhaust particles (DEP), coke (C), pellet ashes (PA), incinerator ashes (IA), and brake dusts (BD). Results may highlight diverse biological activities reflecting the different origins of the PM, and show the ability of the model to detect subtle differences in both genotoxicity and inflammatory potential.

## 2. Materials and Methods

### 2.1. Cell Culture

BEAS-2B cells were purchased from Sigma Aldrich (Darmstadt, Germany, cod. 95102433) and were grown on CELL-BIND^®^ 75 cm^2^ flasks (Corning, New York, NY, USA) at 37 °C, 5% CO_2_ in LHC-9 medium (Gibco, Life Technologies, Monza, Italy) with 1% of penicillin/streptomycin solution (Sigma Aldrich, Darmstadt, Germany). Before experiments, supports were coated with a solution of 0.01% collagen (Sigma Aldrich, Darmstadt, Germany) in Phosphate Buffered Saline (PBS—Fisher Molecular Biology, Rome, Italy) for 2 h at room temperature. This solution was removed, supports were washed with PBS, and then used for cell seeding.

### 2.2. Particulate Matter Samples

As reference PM, NIES certified reference material No. 8 vehicle exhaust particulates DEP was used (Environment Agency NIES, Ibaraki, Japan). Specific details about the collection and chemical characterization of coke (C), pellet ashes (PA), incinerator ashes (IA), and brake dust (BD) are largely reported by Marcoccia et al. [17] and Frezzini et al. [16]. Briefly, C was taken near a refinery plant, PA was produced by pellet burning and collected inside the hood of a domestic stove, IA was sampled by a fine-mesh filter placed in a chimney of a waste-to-energy plant for non-hazardous waste, while BD derived from brake pads linings. All dusts were homogenized and sieved at 50 µm (Giuliani, Torino, Italy) before use. The chemical composition of the soluble and insoluble fraction of the PMs is reported in Supplementary Tables S1 and S2.

### 2.3. Size Distribution and Chemical Composition of PM Samples

We assessed the particle size distribution in each sample on a particle-by-particle basis with Single Particle Extinction and Scattering (SPES), a light scattering technique described in Potenza et al. [26]. Solid dust samples were suspended in deionized, sterile water in a beaker at a concentration of ~10 mg/L, then sonicated and stirred for 60 s. About five minutes elapsed before measurements to allow for the deposition of any large particles (~100 µm and larger); we then withdrew the samples into the instrument with a peristaltic pump at 2 mL/min. As revealed by light scattering data, almost all the particles have a compact shape, with the notable exception of DEP samples that included large aggregates. The diameter distributions were estimated by first inferring the refractive index from the SPES data according to the procedure discussed in Potenza et al. [26]. This analysis gives an estimate of the size of each particle. Effective refractive indexes of non-spherical particles were lower than their corresponding bulk value and even more so for aggregates, as expected [27,28].

As shown in Table 1, C, PA, and IA have modal diameters of 0.3–0.4 µm which are comparable to the smaller of the two modes from the DEP distribution while BD is slightly larger (0.6 µm).

As reported in Tables S1 and S2, among the toxic components present in PM samples, elemental carbon content is relevant in Diesel Exhaust Particles (DEP), coke (C), pellet ashes (PA), and brake dusts (BD), accounting for more than 30% of the total carbon detected in the samples. It is noteworthy that C, PA, and IA are rich in PAHs and other noxious organics species [16,17]. Regarding minor and trace elements, the highest concentration was detected in DEP, PA, and BD [29,30].

### 2.4. Treatments

For all the experiments, PM were weighted, suspended in sterile water at 25 mg/mL, and stored at 4 °C until use. BEAS-2B were seeded and allowed to grow for 48 h before treatments. Cells were exposed to 25–50–100–150 µg/mL PM in LHC-9 for 24 h, where not differently stated. These concentrations were selected because similar ranges of toxicity are generally used to test toxicity in many literature studies on diesel exhaust particles e.g., [31,32]. These concentrations should be considered relevant for human exposure in heavily polluted area, as the use of deposition models suggested that in vitro concentrations of 100 μg/mL may be representative for the amount of particles deposited in the human lungs after 24 h inhalation of ambient air concentrations of 100–150 μg/m^3^ [33,34,35,36].

### 2.5. Cytotoxicity

Cell viability was assessed using the MTT reduction test [37]. Briefly, cells seeded in 96-well plates were exposed in triplicate to increasing concentrations of PM for 24 h. After treatment, cells were washed with PBS and incubated at 37 °C, 5% CO_2_ for 3 h in LHC-9 containing 0.5 mg/mL MTT (Sigma Aldrich, Darmstadt, Germany). Cells were washed with PBS, and formazan solubilized with 100 µL/well of DMSO (Sigma Aldrich, Darmstadt, Germany). Absorbance was read at 595 nm in a microplate reader (Molecular Devices, Emax precision microplate reader, San José, CA, USA). Results are expressed as percentages versus control untreated cells (100%).

### 2.6. Evaluation of Apoptosis

Cells were seeded in 60 mm Petri dishes and exposed to increasing concentrations of PM for 24 h. After treatments, cells, and supernatants were collected and centrifuged for 5 min at 2500 rpm. Apoptosis was evaluated using a commercially available kit following the supplier’s instructions (Alexa Fluor^®^ 488 Annexin V/Dead Cell Apoptosis Kit, Invitrogen, ThermoFisher Scientific, Waltham, MA, USA). Briefly, cells were incubated with Annexin V conjugate with Alexa Fluor^®^ 488 and propidium iodide (PI) for 15 min in Annexin Binding Buffer at room temperature (RT). After incubation, samples were suspended in PBS and read on flow cytometry (Novocyte 3000, ACEA Bioscience, Inc., San Diego, CA, USA). 10,000 cells were analyzed by NovoExpress Software (ACEA Bioscience, Inc., San Diego, USA).

### 2.7. Quantification of Intracellular ROS

Cells were seeded in 96-well black plates with clear and flat bottoms (Brand, Wertheim, Germany), and each treatment was performed in triplicate. Cells were treated with increasing concentrations of PM for 30 min, 1 h, or 4 h. After treatments, the culture medium was removed and replaced with fresh medium containing 25 µM 2′,7′-dichlorodihydrofluorescein diacetate (Sigma Aldrich, Darmstadt, Germany) for 30 min at 37 °C. Cells were washed with PBS and fluorescence was read at 495 nm with Enspire (PerkinElmer, Waltham, USA). Protein quantification was performed using the Lowry method [38] and used to normalize the results. Results are expressed as FU/µg (fluorescence units on micrograms of proteins).

### 2.8. Assessment of Genotoxicity: Evaluation of H2AX Phosphorylation and Micronuclei Detection

The presence of double-strand breaks and micronuclei was performed using immunofluorescence techniques. Cells were seeded on pre-coated 12 mm diameter glass slides in a 24-wells plate. After treatment, cells were fixed in cold methanol at −20 °C for 10 min and cells were permeabilized with 0.5% Triton-X100 in PBS for 10 min. 5% Bovine Serum Albumin (Sigma Aldrich, Darmstadt, Germany) in PBS was used to block unspecific binding sites. Cells were then incubated overnight at 4 °C with the primary antibody (histone H2AX.XS 139ph antibody, 1:500—Active Motif, Waterloo, Belgium), allowing the recognition of the phosphorylation of serine-139 of the H2AX histone. Slides were washed with PBS and immediately incubated with the secondary antibody, conjugated with Alexa Fluor^®^ 488 (AlexaFluor^®^ 488 Goat anti-Rabbit IgG H+L, 1:400—Immunological Sciences, Rome, Italy), for 1 h at RT. Cells were washed with PBS, and finally, slides were mounted with 10 µL of Vectashield^®^ Mounting Medium (containing DAPI—Vector Laboratories, Burlingame, USA). Slides were allowed to dry in order to be read with 100X oil objective with fluorescence microscopy (Axiovert 200M) using DAPI and FITC filters. 100 cells/samples were analyzed to obtain data for the γ-H2AX analysis, while the same samples were used for the evaluation of micronuclei’s presence (1000 cells/sample, following the criteria described by Fenech [39]).

### 2.9. Interleukin-8 (IL-8) Secretion

Cells were seeded in 60 mm Petri dishes and grown to confluence, subsequently, they were treated for 24 h. At the end of the treatment, supernatants were collected and stored at −20 °C until evaluation. The secretion of the pro-inflammatory cytokine IL-8 was assessed by a commercially available kit following the supplier’s instructions (Human Interleukin-8 Development Kit, 900-K18, PeproTech, Cranbury, NJ, USA; sensitivity range 8–1000 pg/mL). Data are expressed in pg/mL for each sample.

### 2.10. Statistical Analysis

Each experiment (*n*) was repeated at least three times. Data are reported as mean ± standard error of the mean. Statistical analysis was performed using the software GraphPad Prism 8.0.2 (GraphPad Software, San Diego, CA, USA). A one-way ANOVA test was chosen for the analysis of all results, in association with Dunnett’s Multiple Comparison post hoc test. Results were considered significant at *p* < 0.05.

## 3. Results

### 3.1. Cell Death

Cells were exposed to increasing PM concentrations (25–50–100–150 µg/mL) for 24 h to assess cell viability using the MTT assay. Results are shown in Table 2., a concentration-dependent reduction in cell viability was observed only for PA, however, it reached statistical significance at 150 µg/mL for PA and IA, and at all the tested concentrations for BD. Even in the absence of a dose response, all doses were effective in reducing cell survival.

**Figure 1:**
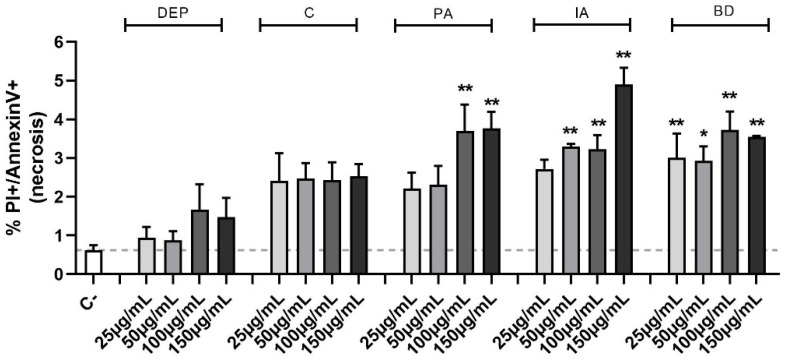
Evaluation of cell viability through Annexin V test in flow cytometry. BEAS-2B cells were treated with 25, 50, 100, or 150 µg/mL of DEP, C, PA, IA, or BD for 24 h. The graph shows the percentage of necrotic cells population, evaluated via Annexin V test in flow cytometry (positive to both PI and Annexin V staining). Results are expressed as mean ± SEM, *n* = 3. Dashed line represents the mean of the control sample data. Statistical analysis was performed using One-Way ANOVA with Dunnett’s post hoc analysis. * *p* < 0.05, ** *p* < 0.01 vs. C-.

To ascertain the nature of the observed cell death, the Annexin V test was used. This method allows the classification of a cell population into three different groups: alive, apoptotic, and necrotic cells. Evaluation of the percentage of necrotic cells (Figure 1) confirmed the MTT cell viability data, highlighting a statistical significance, compared to the control group, at the concentrations of 100 and 150 µg/mL for PA, 50, 100, and 150 µg/mL for IA, as well as at all the concentrations of BD. No differences in apoptotic cell populations were observed among the different treatments and data are not shown.

### 3.2. ROS Production

**Figure 2:**
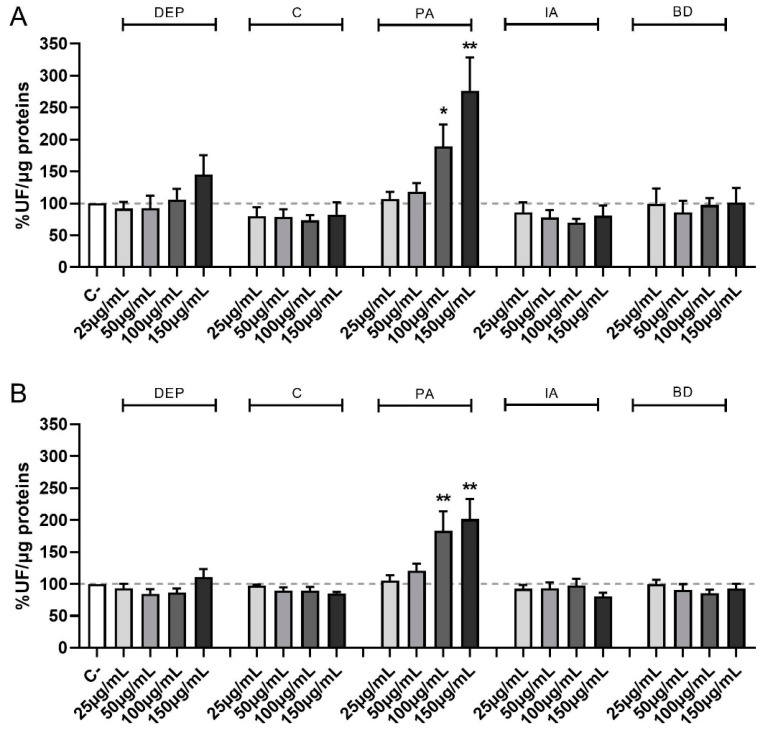
Time course of ROS production following PM exposure. Cells were treated with 25, 50, 100, or 150 µg/mL of DEP, C, PA, IA, or BD for 30 min (**A**) and 1 h (**B**). Results are expressed as the mean of fluorescence ± SEM, in percentage compared to C- (fixed at 100%, dashed line), *n* = 3. Statistical analysis was performed using One-Way ANOVA with Dunnett’s post hoc analysis. * *p* < 0.05, ** *p* < 0.01 vs. C-.

Figure 2 shows the time-course of ROS formation following exposure to increasing concentrations of PM samples at the different time points of treatments evaluated, i.e., 30 min (A) and 1 h (B). PM obtained from different sources show different effects also for this parameter, with the formation of ROS being evident and statistically significant compared to controls only after PA treatment at the concentration of 100 and 150 µg/mL at 30 min, 1 h, and, only at the highest concentration, at 4 h (data not shown).

### 3.3. Histone H2AX Phosphorylation

**Figure 3:**
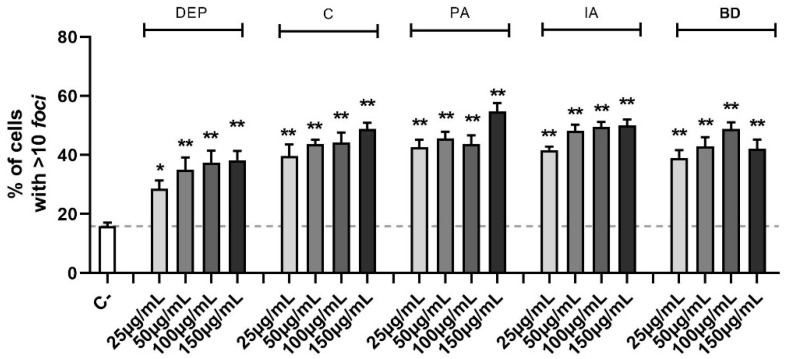
Quantification of DSBs through γ-H2AX evaluation via immunofluorescence. Cells were treated with 25, 50, 100, or 150 µg/mL of DEP, C, PA, IA, or BD for 24 h. The results are expressed as a percentage of cells classified based on the amount of DNA damage in 3 populations: 0–5 *foci*, absence of damage, 6–10 *foci*, medium damage, >10 *foci*, and high damage (shown in the graph). Dashed line represents the mean value of the control. Results are expressed as the mean of percentages ± SEM, *n* = 3. Statistical analysis was performed using One-Way ANOVA with Dunnett’s post hoc analysis. * *p* < 0.05, ** *p* < 0.01 vs. C-.

The identification of double-strand breaks (DSBs) is possible through the study of the phosphorylation of serine-139 on H2AX histone. This is a marker of the DNA repair mechanism, dedicated to the early recognition of DSB sites and evaluable via immunofluorescence. The amount of DNA damage, defined by the number of fluorescent *foci* per cell, is shown in Figure 3. Treatments with all concentrations of the different PM types induced DSB, as shown by the increase of the number of cells with more than 10 *foci* after 24 h in a statistically significant manner (the decrease of cells with less than 5 *foci* was also present but the data are not shown). For the intermediate class (6–10 *foci*), relevant variations were not observed.

### 3.4. Micronuclei Formation

**Figure 4:**
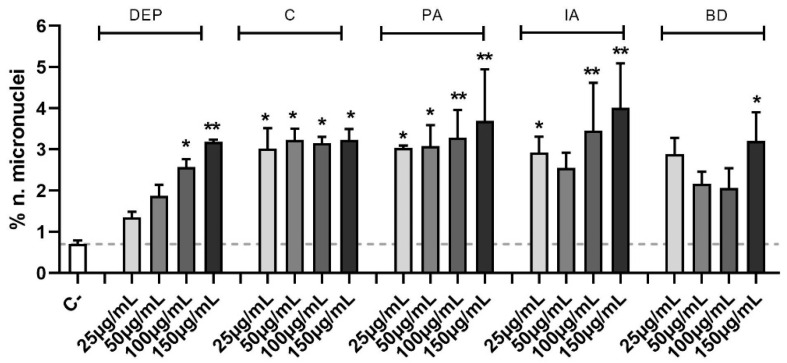
Micronuclei evaluation via immunofluorescence. Cells were treated with 25, 50, 100, or 150 µg/mL of DEP, C, PA, IA, or BD for 24 h. Control sample mean is also reported as a dashed line. Results are expressed as the mean of percentages ± SEM, *n* = 3. Statistical analysis was performed using One-Way ANOVA with Dunnett’s post hoc analysis. * *p* < 0.05, ** *p* < 0.01 vs. C-.

In Figure 4 data are expressed as percentage of micronuclei number over the control. The increase of micronuclei in PM-treated samples compared to controls results to be statistically significant at all PM concentrations, except for 25 and 50 µg/mL DEP, 50 µg/mL IA, and 25, 50, and 100 µg/mL BD. In particular, the genomic insult is strongly induced by PA and IA, which at its highest concentration peaks at 4.0 ± 1.1% of micronuclei.

### 3.5. Secretion of the Pro-Inflammatory Cytokine IL-8

**Figure 5:**
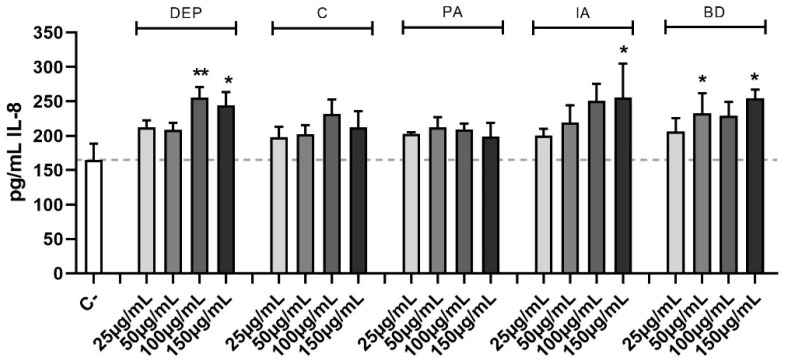
Secretion of IL-8. Cells were treated with 25, 50, 100, or 150 μg/mL of DEP, C, PA, IA, or BD for 24 h. Dashed line represents the mean of the control sample. Results are expressed as the mean of pg/mL ± SEM, *n* = 4. Statistical analysis was performed using One-Way ANOVA with Dunnett’s post hoc analysis. * *p* < 0.05, ** *p* < 0.01 vs. C-.

The effect of different PM samples on IL-8 release is shown in Figure 5. DEP induced the release of IL-8 at the highest concentrations, while among the other PM types, only IA and BD caused a statistically significant secretion of IL-8.

## 4. Discussion

The purpose of this study was to investigate the effects of different PM types on cell viability, oxidative stress, DNA damage, and release of a pro-inflammatory mediator in the human bronchial cell line BEAS-2B.

Five different types of PM from various emission sources were tested in parallel. Several studies have attributed toxicological endpoints to BD by demonstrating that brake wear particles, rich in metals such as Fe and Cu, can damage cells determining pro-inflammatory cell response, thus triggering oxidative stress reactions [29,40,41]. DEP has been for years one of the main topics of investigations on traffic-derived particulate toxicity [31,32,42]. The toxicity of DEP is mainly related to its small size that permits it to penetrate tissues, coupled with the capability of diesel-combustion derived particles to absorb toxic compounds on their surface, such as complex mixtures of organic compounds [43,44,45]. In vivo and in vitro experiments showed significant evidence of the activation of inflammatory cascades and redox-sensitive signaling pathways [46]. Biomass combustion-derived particles, such as the PA used in this work, have emerged as capable of stimulating adverse effects in biological systems, causing DNA damage, as well as altering the cell cycle [20,47]. This is due to the presence of several toxic compounds, such as PAHs and trace elements that are linked to various genotoxic and carcinogenic effects [48,49]. Biomass burning-related PM is mainly composed of organic fractions, and it contains high concentrations of organic species which have been considered responsible for genotoxic and oxidative stress effects [29]. Lastly, IA from a waste-to-energy plant for non-hazardous waste is known to contain a variety of organic compounds, including PAHs, as well as non-negligible concentrations of heavy metals, such as Cd, Cu, Pb, and Zn [16,50]. IARC reported some of the compounds present in biomass burning PM emissions to be carcinogenic and mutagenic [51]; consequently, IA can exert adverse biological effects [52,53,54]. At high concentrations, a decrease in cell viability was observed, and among the different death pathways, our results indicate necrosis as the most represented whereas no indication of apoptosis was observed, confirming the previous data reported in the literature [55,56,57]. The cell death observed, in particular, caused by BD and the highest concentration of PA and IA, is likely due to the high concentration of heavy metals. Literature data indicate Mn and Cd as heavily cytotoxic for human lung epithelium [58,59]. The chemical characterization of the PM tested [29] indicates Mn as one of the main components of PA, while Cd is present in BD at a concentration higher than 2 g/kg.

The chemical characterization of PM from different sources is useful, not only for the quantification of heavy metals, but also for the identification of other toxicants, such as PAHs and redox cycling quinones [60,61]. Indeed, the literature reported their ROS-generating abilities by undergoing redox recycling and reducing oxygen to produce superoxide radicals [62,63]. In the previous chemical characterization of the PM tested in the current study, the oxidative potential was also assessed using several acellular methods, such as dithiothreitol, ascorbic acid, and 2′,7′-dichlorofluorescin assays [30]. The highest oxidative potential was observed for PA and the lowest for C and IA, the results obtained in the present study confirm this capability. However, Piacentini et al. [30] also found BD to have oxidative potential, which was not confirmed in our work. This could be explained by the different time elapsed between sample collection and oxidative potential analysis, that occurred much later for the samples analyzed in this study, suggesting that the same components, whose stability and bioavailability change in time, can show a reduced oxidative potential.

The ability of PM to induce DNA damage, as breaks and genomic aberrations, is well known [64]. Two of the most commonly evaluated biomarkers of DNA damage are the phosphorylation of H2AX on serine 139 and the formation of micronuclei [65,66]. These lesions may be induced by ROS damaging DNA strands, and ultimately generating adducts and breaks [67,68]. Although the production of ROS is elicited only with PA treatment, all the tested PM samples have been found able to induce genotoxic insult on BEAS-2B, suggesting a direct effect on the DNA strand not directly linked to ROS production. The amount of damage recognized by γ-H2AX increases in a dose-dependent manner in all PM samples tested. The PM mutagenic potential has been demonstrated and confirmed through the evaluation of micronuclei, with C, PA, and IA being the most effective [69,70].

Inflammation is another well-described effect associated with PM exposure, mainly resulting from the presence of metals, PAHs, and microorganisms [12]. In the current study, the PM samples tested showed a different pro-inflammatory potential, with DEP, IA, and BD being the most effective in inducing IL-8 secretion. Studies demonstrated that IL-8 secretion could be induced by PAHs [20,46], typically present at a high level in DEP and IA, which can explain the secretion. While for BD, which does not contain high levels of PAHs, the presence of Cu could be responsible for the relevant induction of IL-8 secretion observed [71].

## 5. Conclusions

The present study showed how PM originating from different sources is characterized by a diverse biological activity. All the investigated samples have a significant genotoxic and mutagenic effect on the BEAS-2B cell line, which does not appear to be correlated with oxidative stress, but rather with the different chemical composition. In terms of environmental hazardousness, due to the mixture of atmospheric particulate matter emitted from a variety of sources, every source has a noxious effect but with a different genotoxic and pro-inflammatory profile in relation to both particle size and chemical composition. However, it is interesting to note that all samples showed a unimodal size distribution (apart from DEP, where large aggregates were also observed) with modes in the submicron range and BD was the sample with a slightly higher mode (at about 0.6 µm).

BD resulted to be the most cytotoxic, as suggested by the presence of a high concentration of heavy metals, in particular Fe and Cd. Several studies in the literature have shown that transition metals, such as Fe, Cd, Ni, and Cu, may undergo the Fenton reaction and initiate signaling pathways leading to ROS production [72,73,74] and we also showed the increase of ROS production in PA-treated samples; among the PM evaluated in our study it is the one that contained more of these metals. IA appears to be the most dangerous for bronchial cells: it is active in the generation of genotoxic damage, cytotoxicity, and inflammation induction. This is due to its chemical composition where PAHs and heavy metals are abundant.

Overall, this study indicates that the BEAS-2B model represents a valuable tool for screening the biological activity of PM originating from different sources, able to detect subtle differences.

## Figures and Tables

**Table 1:** Overview of the main parameters of the diameter distributions, sampled with a base 10 logarithmic binning.

Sample	Mode [µm]	Std Dev [µm]	Min [µm]	Max [µm]
DEP	0.4; 6.0 (bimodal)	2.4	0.2	10.6
Coke (C)	0.3	0.4	0.2	2.4
Pellet ash (PA)	0.4	0.3	0.3	2.4
Incinerator ash (IA)	0.4	0.6	0.3	3.1
Brake dust (BD)	0.6	0.6	0.2	4.4

**Table 2:** Evaluation of cell viability through MTT test.

Sample	-	25 µg/mL	50 µg/mL	100 µg/mL	150 µg/mL
Control	100.0 ± 0.0				
DEP		98.3 ± 12.4	76.0 ± 10.9	79.7 ± 6.2	74.3 ± 10.2
Coke (C)		83.0 ± 10.7	84.0 ± 6.4	90.7 ± 4.8	88.3 ± 5.8
Pellet ash (PA)		85.3 ± 2.8	84.7 ±1.2	82.7 ± 6.1	73.9 ± 10.6 *
Incinerator ash (IA)		76.3 ± 7.6	87.3 ± 7.8	84.7 ± 5.2	65.0 ± 12.0 *
Brake dust (BD)		76.0 ± 3.2 *	69.0 ± 8.7 **	76.0 ± 5.5	58.7 ± 9.1 **

BEAS-2B cells were treated with 25, 50, 100, or 150 µg/mL of DEP, C, PA, IA, or BD for 24 h. In Table 2 results obtained with MTT assay are reported. Data are expressed as a percentage of viable cells normalized on the control samples (100%). Results are expressed as mean ± SEM, *n* = 3. Statistical analysis was performed using One-Way ANOVA with Dunnett’s post hoc analysis. * *p* < 0.05, ** *p* < 0.01 vs. C-.

## Data Availability

Data are available from the corresponding author upon request.

## Supplementary Materials

The following supporting information can be downloaded at: https://www.mdpi.com/article/10.3390/toxics11050413/s1, Table S1: Chemical composition of the soluble fraction (mean and standard deviation; six replicates); Table S2: Chemical composition of the insoluble fraction (mean and standard deviation; six replicates).
