# Supplementary material for: Toxicological Profile of PM from Different Sources in the Bronchial Epithelial Cell Line BEAS-2B
Source: Toxics. 2023 Apr 26;11(5):413. doi: 10.3390/toxics11050413 (PMC10222988; doi:10.3390/toxics11050413)
Supplement: Supplementary file 1 [file toxics-11-00413-s001.zip › toxics-2338598-supplementary.pdf]

Table S1. Chemical composition of the soluble fraction (mean and standard deviation; six replicates)

		BD			C			IA			PA		
**UoM**		**Mean**	±	*SD*	**Mean**	±	*SD*	**Mean**	±	*SD*	**Mean**	±	*SD*
mg/Kg	**As**	**1.13**	±	*0.05*	**0.60**	±	*0.03*	**0,74**	±	*0,04*	**2.9**	±	*0.3*
g/Kg	**Ca**	**27.9**	±	*0.4*	**1.29**	±	*0.02*	**8,1**	±	*0,2*	**74**	±	*1*
mg/Kg	**Cd**	**0.30**	±	*0.02*	**0.02**	±	*0.01*	**0,050**	±	*0,002*	**13**	±	*1*
mg/Kg	**Ce**	**0.106**	±	*0.005*	**0.022**	±	*0.005*	**0,30**	±	*0,03*	**0.041**	±	*0.002*
mg/Kg	**Co**	**1.06**	±	*0.00*	**0.05**	±	*0.00*	**0,23**	±	*0,02*	**4.3**	±	*0.3*
mg/Kg	**Cr**	**24.7**	±	*0.4*	**0.83**	±	*0.01*	**2,0**	±	*0,3*	**4.6**	±	*0.1*
mg/Kg	**Cs**	**0.210**	±	*0.001*	**0.010**	±	*0.003*	**0,048**	±	*0,004*	**2.7**	±	*0.2*
mg/Kg	**Cu**	**765**	±	*2*	**13**	±	*2*	**16**	±	*2*	**37**	±	*6*
g/Kg	**Fe**	**6.4**	±	*0.1*	**1.0**	±	*0.3*	**131**	±	*14*	**0.02**	±	*0.02*
mg/Kg	**Mn**	**119.7**	±	*0.3*	**6.4**	±	*0.1*	**1,5**	±	*0,9*	**1751**	±	*24*
mg/Kg	**Mo**	**3.80**	±	*0.01*	**0.827**	±	*0.003*	**0,80**	±	*0,09*	**3.6**	±	*0.2*
mg/Kg	**Ni**	**3.97**	±	*0.01*	**1.88**	±	*0.04*	**0,61**	±	*0,08*	**9**	±	*1*
mg/Kg	**Pb**	**5.94**	±	*0.02*	**9.85**	±	*0.1*	**2,1**	±	*0,1*	**0.42**	±	*0.02*
mg/Kg	**Rb**	**5.6**	±	*0.1*	**0.6**	±	*0.1*	**2,6**	±	*0,2*	**310**	±	*26*
mg/Kg	**Sb**	**13.9**	±	*0.4*	**0.16**	±	*0.03*	**5,2**	±	*0,6*	**1.6**	±	*0.2*
mg/Kg	**Sn**	**1.62**	±	*0.03*	**0.130**	±	*0.002*	**0,54**	±	*0,04*	**0.46**	±	*0.04*
mg/Kg	**Sr**	**98**	±	*3*	**4**	±	*3*	**35**	±	*4*	**542**	±	*29*
mg/Kg	**Ti**	**0.98**	±	*0.02*	**13**	±	*1*	**1,8**	±	*0,1*	**0.061**	±	*0.001*
mg/Kg	**Tl**	**0.04**	±	*0.01*	**0.002**	±	*0.001*	**0,0020**	±	*0,0002*	**1.05**	±	*0.05*
mg/Kg	**V**	**1.29**	±	*0.04*	**1.08**	±	*0.01*	**1,2**	±	*0,1*	**3.7**	±	*0.4*
mg/Kg	**Zn**	**2120**	±	*8*	**100**	±	*5*	**10**	±	*4*	**525**	±	*27*
g/kg	**NO_2^-**	**<0.1**			**<0.1**			**<0.1**			**6.5**	±	*0.3*
g/kg	**NO_3^-**	**4.0**	±	*0.4*	**<0.1**			**<0.1**			**2.2**	±	*0.3*
g/Kg	**SO_4^{--}**	**5.9**	±	*0.2*	**<0.1**			**2.1**	±	*0.4*	**37**	±	*1*
g/kg	**WSOC**	**5.7**	±	*0.1*	**9.5**	±	*0.2*	**5.3**	±	*0.2*	**22**	±	*1*

Table S2. Chemical composition of the insoluble fraction (mean and standard deviation; six replicates)

UoM		BD Mean	±	SD	C Mean	±	SD	IA Mean	±	SD	PA Mean	±	SD
mg/Kg	**As**	**18.0**	±	*0.1*	**0.52**	±	*0.04*	**8,1**	±	*0,4*	**2.3**	±	*0.3*
g/Kg	**Ca**	**12.9**	±	*0.3*	**1.38**	±	*0.03*	**-**			**74**	±	*2*
mg/Kg	**Cd**	**0.73**	±	*0.08*	**0.05**	±	*0.04*	**6,3**	±	*0,4*	**20**	±	*3*
mg/Kg	**Ce**	**26.2**	±	*0.2*	**0.3**	±	*0.1*	**20**	±	*1*	**9**	±	*1*
mg/Kg	**Co**	**13.9**	±	*0.5*	**1.02**	±	*0.04*	**25**	±	*1*	**9**	±	*1*
mg/Kg	**Cr**	**3083**	±	*74*	**9.6**	±	*0.2*	**236**	±	*11*	**20.4**	±	*0.5*
mg/Kg	**Cs**	**2.99**	±	*0.03*	**0.04**	±	*0.01*	**1,00**	±	*0,05*	**0.43**	±	*0.05*
mg/Kg	**Cu**	**4286**	±	*17*	**43**	±	*11*	**1281**	±	*64*	**204**	±	*53*
g/Kg	**Fe**	**198**	±	*5*	**16**	±	*4*	**23**	±	*1*	**3.7**	±	*0.1*
mg/Kg	**Mn**	**1093**	±	*4*	**40**	±	*1*	**650**	±	*29*	**17869**	±	*364*
mg/Kg	**Mo**	**171**	±	*1*	**75.5**	±	*0.1*	**7,2**	±	*0,4*	**1.2**	±	*0.1*
mg/Kg	**Ni**	**108**	±	*3*	**355**	±	*1*	**160**	±	*8*	**28**	±	*4*
mg/Kg	**Pb**	**677**	±	*4*	**8.9**	±	*0.1*	**529**	±	*23*	**55**	±	*5*
mg/Kg	**Rb**	**27**	±	*1*	**0.5**	±	*0.1*	**13**	±	*0,6*	**31**	±	*4*
mg/Kg	**Sb**	**292**	±	*1*	**4.07**	±	*0.01*	**30**	±	*1,5*	**1.7**	±	*0.3*
mg/Kg	**Sn**	**1419**	±	*3*	**14.7**	±	*0.3*	**83**	±	*4*	**21**	±	*3*
mg/Kg	**Sr**	**159**	±	*7*	**6**	±	*7*	**278**	±	*13*	**717**	±	*57*
mg/Kg	**Ti**	**526**	±	*13*	**894**	±	*21*	**1728**	±	*85*	**18.9**	±	*0.5*
mg/Kg	**Tl**	**0.3**	±	*0.1*	**0.03**	±	*0.01*	**0,059**	±	*0,002*	**0.9**	±	*0.1*
mg/Kg	**V**	**7.6**	±	*0.4*	**557**	±	*1*	**27**	±	*1*	**8**	±	*1*
mg/Kg	**Zn**	**3197**	±	*18*	**160**	±	*12*	**1,8**	±	*0,1*	**1157**	±	*91*
g/kg	**EC**	**17**	±	*1*	**310**	±	*15*	**23**	±	*3*	**44**	±	*4*
g/kg	**WIOC**	**30**	±	*3*	**146**	±	*5*	**41**	±	*3*	**4**	±	*1*
